# Feature Selection Method Based on Artificial Bee Colony Algorithm and Support Vector Machines for Medical Datasets Classification

**DOI:** 10.1155/2013/419187

**Published:** 2013-07-28

**Authors:** Mustafa Serter Uzer, Nihat Yilmaz, Onur Inan

**Affiliations:** ^1^Electrical-Electronics Engineering, Faculty of Engineering, Selcuk University, Konya, Turkey; ^2^Computer Engineering, Faculty of Engineering, Selcuk University, Konya, Turkey

## Abstract

This paper offers a hybrid approach that uses the artificial bee colony (ABC) algorithm for feature selection and support vector machines for classification. The purpose of this paper is to test the effect of elimination of the unimportant and obsolete features of the datasets on the success of the classification, using the SVM classifier. The developed approach conventionally used in liver diseases and diabetes diagnostics, which are commonly observed and reduce the quality of life, is developed. For the diagnosis of these diseases, hepatitis, liver disorders and diabetes datasets from the UCI database were used, and the proposed system reached a classification accuracies of 94.92%, 74.81%, and 79.29%, respectively. For these datasets, the classification accuracies were obtained by the help of the 10-fold cross-validation method. The results show that the performance of the method is highly successful compared to other results attained and seems very promising for pattern recognition applications.

## 1. Introduction

Pattern recognition and data mining are the techniques that allow for the acquirement of meaningful information from large-scale data using a computer program. Nowadays, these techniques are extensively used, particularly in the military, medical, and industrial application fields, since there is a continuously increasing amount and type of data in these areas, due to advanced data acquisition systems. For this reason, for the obtained data set, data reduction algorithms are needed for filtering, priority sorting, and providing redundant measurements to detect the feature selection. By using these algorithms, quality data is obtained, which in turn raises the quality of the analyzing systems or the success of the recognition systems. In particular, medical applications with ever-increasing popularity and use of advanced technology are the most important field in which these algorithms are used. Many new algorithms developed in the field of medicine are tested on the disease data presented for the common use of all the scientists, and their performances are compared. The datasets from UCI database are very popular for this purpose. The algorithm developed and tested on hepatitis, liver disorders, and diabetes data from UCI was compared with studies in the literature that use the same datasets. These data sets consist of diseases that are commonly encountered in society and significantly reduce the quality of life of patients. The selected data sets are comprised of a variety of test and analysis device data and personal information about the patients. The main objective our work is the integration of the developed systems to these test and analysis devices and to provide a fully automatic assistance to the physician in the creation of diagnosis systems for the diseases. The diagnosis systems, which can be easily used during routine controls, will make the timely information and the early treatment of patients possible. 

For the dataset recognition aiming diagnosis of the diseases, we propose a two-stage approach. The first stage has used the clustering with ABC algorithm as selection criteria for feature selection, and, thus, more effective feature selection methods have been constituted. Hence, it has been made possible both to select the related features faster and to reduce the feature vector dimensions. In the second stage, the reduced data was given to the SVM classifier and the accuracy rates were determined. The *k*-fold cross-validation method was used for improving the classifier reliability. The datasets we have worked on have been described in the Background section. As it is seen from the results, the performance of the proposed method is highly successful compared to other results attained and seems very promising for pattern recognition applications.

### 1.1. Background

The developed approach has been tested for the diagnosis of liver diseases and diabetes, which are commonly seen in the society and both reduce the quality of life. In the developed system, the hepatitis and liver disorders datasets were used for the diagnosis of liver disease, and the Diabetes dataset was used for the diagnosis of diabetes.

The liver disease diagnostics studies using the Hepatitis dataset were as follows: Polat and Güneş [1] proposed a new diagnostic method of hepatitis disease based on a hybrid feature selection (FS) method and artificial immune recognition system (AIRS) using fuzzy resource allocation mechanism. The obtained classification accuracy of the proposed system was 92.59%. A machine learning system studied by Polat and Güneş [2] was conducted to identify hepatitis disease. At first, the feature number of dataset on hepatitis disease was reduced from 19 to 10 by using in the feature selection (FS) subprogram and C4.5 decision tree algorithm. Then, fuzzy weighted preprocessing was used for weighting the dataset after normalizing between 0 and 1. AIRS classifier system was used while classifying the weighted input values. The classification accuracy of their system was 94.12%. Principal component analysis (PCA) and artificial immune recognition system (AIRS) were conducted for hepatitis disease prediction in the study by Polat and Güneş [3]. Classification accuracy, 94.12%, was obtained with the proposed system using 10-fold cross-validation. A method which had an accuracy value of 96.8% for hepatitis dataset was proposed by Kahramanli and Allahverdi [4], and in this method extracting rules from trained hybrid neural network was presented by using artificial immune systems (AISs) algorithm. An automatic diagnosis system using linear discriminant analysis (LDA) and adaptive network based on fuzzy inference system (ANFIS) was proposed by Dogantekin et al. [5] for hepatitis diseases. This automatic diagnosis system of hepatitis disease diagnostics was obtained with a classification accuracy of about 94.16%. Bascil and Temurtas [6] realized a hepatitis disease diagnosis based on a multilayer neural network structure that used the Levenberg- Marquardt algorithm as training algorithm for the weights update with a classification accuracy of 91.87% from 10-fold cross-validation. 

The studies for the diagnosis of liver disease in using the liver disorders dataset were as follows: by Lee and Mangasarian [7], smoothing methods were applied to generate and solve an unconstrained smooth reformulation of the support vector machine for pattern classification using a completely arbitrary kernel. They termed such reformulation a smooth support vector machine (SSVM). Correct classification rate of the proposed system with CV-10 was 70.33% for liver disorders dataset. In Van Gestel et al.'s [8] article, the Bayesian evidence framework was combined with the LS-SVM classifier formulation. Correct classification rate of proposed system with CV-10 was 69.7% for liver disorders dataset. Gonçalves et al. [9] a new neuro-fuzzy model, especially created for record classification and rule extraction of databases, named as inverted hierarchical neuro-fuzzy BSP System (HNFB). Correct classification rate of this system was 73.33% for liver disorders dataset. Özşen and Güneş [10] aimed to contribute to an artificial immune system AIS by attaching this aspect and used the Euclidean distance, Manhattan distance, and hybrid similarity measure with simple AIS. Correct classification rate of the proposed system with AWAIS was 70.17%, with hybrid similarity measure 60.57%, with the Manhattan distance 60.21%, with the Euclidean distance 60.21% for liver disorders. Li et al. [11] proposed a nonlinear transformation method based on fuzzy to find classification information in the original data attribute values for a small dataset and used a support vector machine (SVM) as a classifier. Correct classification rate of the proposed system was 70.85% for liver disorders. Chen et al. [12] proposed an analytical approach by taking an integration of particle swarm optimization (PSO) and the 1-NN method. Correct classification rate of proposed system with 5-fold cross-validation was 68.99% for liver disorders dataset. A hybrid model based on integrating a case-based reasoning approach and a particle swarm optimization model were proposed by Chang et al. [13] for medical data classification.

Another disease that we selected is diabetes. Some of the most important studies conducted on this dataset are as follows: Şahan et al. [14] proposed attribute weighted artificial immune system (AWAIS) with weighting attributes due to their important degrees in class discrimination and using them for the Euclidean distances calculation. AWAIS had a classification accuracy of 75.87 using 10-fold cross-validation method for diabetes dataset. Polat and Güneş [15] worked on diabetes disease using principal component analysis (PCA) and adaptive neuro-fuzzy inference system (ANFIS). The obtained test classification accuracy was 89.47% by using the 10-fold cross-validation. Polat et al. [16] proposed a new learning system which is cascade and used generalized discriminant analysis and least square support vector machine. The classification accuracy was obtained as 82.05%. Kahramanli and Allahverdi [17] presented a hybrid neural network that achieves accuracy value of 84.24% using artificial neural network (ANN) and fuzzy neural network (FNN) together. Patil et al. [18] proposed hybrid prediction model (HPM) which uses Simple *k*-means clustering algorithm for verifying the chosen class labels and then using the classification algorithm on the result set. Accuracy value of HPM was 92.38%. Isa and Mamat [19] presented a modified hybrid multilayer perceptron (HMLP) network for improving the conventional one, and the average correct classification rate of the proposed system was 80.59%. Aibinu et al. [20] proposed a new biomedical signal classification method using complex-valued pseudo autoregressive (CAR) modeling approach. The presented technique obtained a classification accuracy of 81.28%. 

## 2. Preliminaries

### 2.1. Feature Selection

Feature selection provides a smaller but more distinguishing subset compared to the starting data, selecting the distinguishing features from a set of features and eliminating the irrelevant ones. Reducing the dimension of the data is aimed by finding a small important features set. This results in both reduced processing time and increased classification accuracy. 

The algorithm developed in this study was based on the sequential forward selection (SFS) algorithm, which is popular in these algorithms. SFS is a method of feature selection offered by Whitney [21]. Sequential forward selection is the simplest greedy search algorithm which starts from the empty set and sequentially adds the feature *x* ^+^ for obtaining results in the highest objective function *J*(*Y*_*k*_ + *x* ^+^) when combined with the features *Y*_*k*_ that have already been selected. Pseudo code is given Pseudocode 1 for SFS [22].

**Pseudocode 1:**
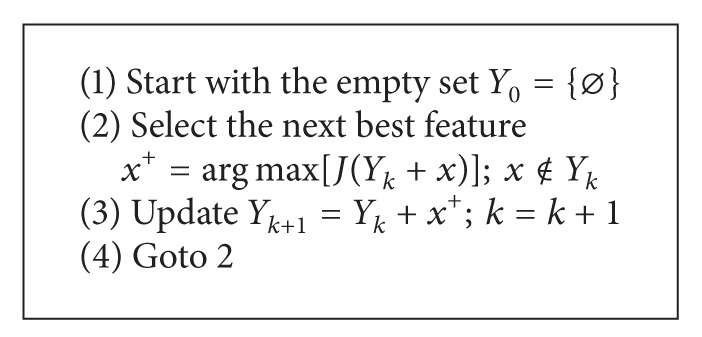
Pseudo code for SFS [22].

In summary, SFS begins with zero attributes and then evaluates the whole feature subsets with only one feature, and the best performing one adds this subset to the best performing feature for subsets of the next larger size. This cycle repeats until there is no improvement in the current subset [23].

The objection function is critical for this algorithm. Finding the highest value of this function is an optimization problem. Clustering is an ideal method for the detection of feature differentiation. The developed method can be summarized using the ABC algorithm for feature selection aiming clustering problem adaptation. 

#### 2.1.1. Clustering with Optimization

Clustering is a grouping process running on the multi-dimentional data by using similarities. Distance criteria are used to evaluate similarities in samples set. Clustering problems can be expressed as the placement of every object into one *K* cluster for a given *N* number of objects and minimizing the sum of squares of the Euclidean distances between the centers of these objects in the cluster to which they belong. The function that uses the clustering algorithm is given in (1) [24] for minimizing:

(1) $$\begin{array}{c} J\left(w,z\right)=\sum_{i=1}^{N}\;\sum_{j=1}^{K}w_{ij}{||x_{i}-z_{j}||}^{2}. \end{array}$$

Here, *N* is the number of samples, *K* is the number of clusters, *x*_*i*_  (*i* = 1,…, *N*) is the place of the *i*th sample, and the center of the *j*th sample *z*_*j*_  (*j* = 1,…, *N*) can be obtained by (2):

(2) $$\begin{array}{c} z_{j}=\frac{1}{N_{j}}\;\sum_{i=1}^{N}w_{ij}x_{i}. \end{array}$$

Here, *N*_*j*_ is the number of samples in the *j*th cluster, and *w*_*ij*_ is the relationship of *j* cluster and *x*_*i*_ sample with a value of 1 or 0. If the sample *i* (*x*_*i*_) belongs to the *j* cluster, *w*_*ij*_ is 1, otherwise that it is 0.

The clustering process that separates objects into groups can be performed by supervised or unsupervised learning. Training data in unsupervised clustering (also known as automatic clustering) does not need to set class tags. In supervised clustering, however, it should be specified so that the classes can learn the tags. In this study, the datasets used should contain class information since supervised clustering was used. Therefore, the optimization aims to find the centers of clusters by making the objective function minimize, which is the total of the samples distances to centers [24]. In this study, the sum of distances between all training cluster samples and the cluster center (*p*_*i*_^CL_known_(*x*_*j*_)^) that samples belong to in the *n*-dimensional Euclidean space are minimized for adaptation [24]. Consider the following:

(3) $$\begin{array}{c} f_{i}=\frac{1}{D_{\text{Train}}}\sum_{j=1}^{D_{\text{Train}}}d\left(x_{j},p_{i}^{\text{C}{\text{L}}_{\text{known}}(x_{j})}\right). \end{array}$$

Here, *D*_Train_ is the number of training samples, and the total expression in the cost function is for normalizing the number to a value between 0.0 and 1.0. The *p*_*i*_^CL_known_(*x*_*j*_)^ value indicates the center of the class that belongs to the sample that is used according to training data. Here, the ABC algorithm was chosen as the optimization method for clustering. Thus, ABC, as a new clustering method, can also be used in the feature selection algorithms.

### 2.2. Artificial Bee Colony (ABC) Algorithm

Artificial bee colony (ABC) algorithm, as a population-based stochastic optimization proposed by Karaboga in [24–26], realize the intelligent foraging behavior of honey bee swarms. It can be used for classification, clustering and optimization studies. Pseudocode of the ABC algorithm is given as Pseudocode 2.

**Pseudocode 2:**
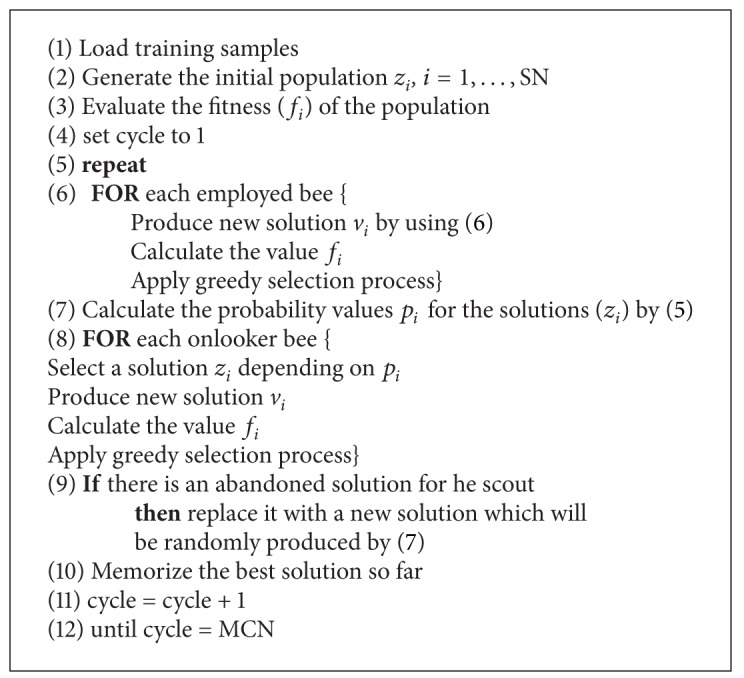
Pseudo-code of the ABC algorithm [24].

An artificial group of bees in the ABC algorithm consists of three different groups: employed bees, onlooker bees, and scout bees. In this algorithm, the number of bees employed in the colony also equals the number of onlooker bees. Additionally, the number of employed bees or onlooker bees equals the number of solutions in the population. An onlooker bee is the bee that waits in the dance area to make the food source selection decision. An onlooker bee is named employed bee once it goes to a food source. An employed bee that has consumed the food source turns into a scout bee, and its duty is to perform a random search to discover new resources. Food supply position—which represents the solution to the optimization problem—and the amount of nectar in the food source depends on the quality of the associated solution. This value is calculated in (4).

(4) $$\begin{array}{c} \text{fi}{\text{t}}_{i}=\frac{1}{1+f_{i}} \end{array}$$

SN in the algorithm indicates the size of the population. At first, the ABC algorithm produces a distributed initial population *P* (*C* = 0) of SN solutions (food source positions) randomly, where SN means the size of population. Each *z*_*i*_ solution is a *D*-dimensional vector for *i* = 1,2, 3,…, SN. Here, *D* is the numbers of cluster products and input size for each dataset. After startup, an investigation is repeated on employed bees, onlooker bees, and scout bees processes until the number of population of positions (*C* = 1,2,…, MCN) is completed. Here, MCN is the maximum cycle number. 

An employed bee makes a small change in position due to the local knowledge in its memory, and a new source is generated. This bee makes a comparison of the nectar amount (fitness amount) of a new source with the nectar amount of previous source and decides which one is higher. If the new position is higher than the old one then it is assimilated into its memory and the old one is forgotten. Otherwise, the position of the previous one stays in its memory. All employed bees that complete the task of research share the position and nectar food source information with the onlooker bees that are in the dance area.

An onlooker bee evaluates the nectar information of all employed bees and chooses a food source depending on the probability of the nectar amount. This probability value (*p*_*i*_) is calculated in (5). Just like the employed bees, the onlooker bee modifies the situation from memory and it checks the nectar amount of the candidate source. If its nectar amount is higher than the previous one and the new position is assimilated into memory and the old one is forgotten, then

(5) $$\begin{array}{c} p_{i}=\frac{\text{fi}{\text{t}}_{i}}{\sum_{n=1}^{\text{SN}}\text{fi}{\text{t}}_{n}}, \end{array}$$

where SN is the number of food sources which is equal to the number of employed bees and the fitness of the fit_*i*_ solution given in (4). The *f*_*i*_ given in (3) is the cost function of the cluster problem. ABC uses (6) for producing a candidate food position:

(6) $$\begin{array}{c} v_{ij}=z_{ij}+\phi_{ij}\left(z_{ij}-z_{kj}\right). \end{array}$$

Here, *k* ∈ {1,2,…, SN}  and *j* ∈ {1,2,…, *D*} are randomly selected indexes. *k* is a random value different from *i*. *ϕ*_*ij*_ is a random number between [−1, 1] which controls the production of neighboring food sources around *z*_*ij*_ and represents comparison of two food sources to a bee.

While onlooker and employed bees perform exploitation in the search area, scout bees control the discovery process and replace the consumed nectar food source with a new food source in the ABC algorithm. If the position cannot be improved as a previously determined cycle number, this food source is accepted as abandoned. The previously determined cycle number is defined as the “*limit*” for abandonment. In this case, there are three control parameters in ABC: the number of food sources (SN) which is equal to the number of employed and onlooker bees, the maximum cycle number (MCN), and the limit value.

If an abandoned source is assumed to be *z*_*i*_ and *j* ∈ {1,2,…, *D*}, the scout looks for a new source to replace *z*_*i*_. This process is described by (7):

(7) $$\begin{array}{c} z_{i}^{j}=z_{\min}^{j}+\text{rand}\left(0,1\right)\left(z_{\max}^{j}-z_{\min}^{j}\right). \end{array}$$

After (*v*_*ij*_) which is each candidate position is produced, the position is evaluated by ABC and its performance is compared with previous one. The performance is compared with the previous one. If the new food source has an equal amount or more nectar than the old one, the new one takes place instead of the old food source in memory. Otherwise, the old one stays in its place in memory. So a greedy selection mechanism is used to make selections among the old source and one of the candidates.

### 2.3. Support Vector Machines (SVMs)

SVM is an effective supervised learning algorithm used in classification and regression analyses for applications like pattern recognition, data mining, and machine learning application. SVM was developed in 1995 by Cortes and Vapnik [27]. Many studies have been conducted on SVM: a flexible support vector machine for regression, an evaluation of flyrock phenomenon based on blasting operation by using support vector machine [28, 29].

In this algorithm, there are two different categories separated by a linear plane. The training of the algorithm is determining the process for the parameters of this linear plane. In multiclass applications, the problem is categorized into groups as belonging either to one class or to others. SVM's use in pattern recognition is described below. 

An *n*-dimensional pattern (object) *x* has *n* coordinates, *x* = (*x*_1_, *x*_2_, …, *x*_*n*_), where each *x* is a real number, *x*_*i*_ ∈ *R* for *i* = 1, 2,…, *n*. Each pattern *x*_*j*_ belongs to a class *y*_*j*_ ∈ {−1, +1}. Consider a training set *T* of *m* patterns together with their classes, *T* = {(*x*_1_, *y*_1_), (*x*_2_, *y*_2_), …, (*x*_*m*_, *y*_*m*_)}. Consider a dot product space *S*, in which the patterns *x* are embedded, *x*_1_, *x*_2_, …, *x*_*m*_ ∈ *S*. Any hyperplane in the space *S* can be written as

(8) $$\begin{array}{c} \left\{x\in S\;|\;w\cdot x+b=0\right\},\;w\in S,\;\;b\in R. \end{array}$$

The dot product *w* · *x* is defined by

(9) $$\begin{array}{c} w\cdot x=\sum_{i=1}^{n}w_{i}x_{i}. \end{array}$$

**Figure 1:**
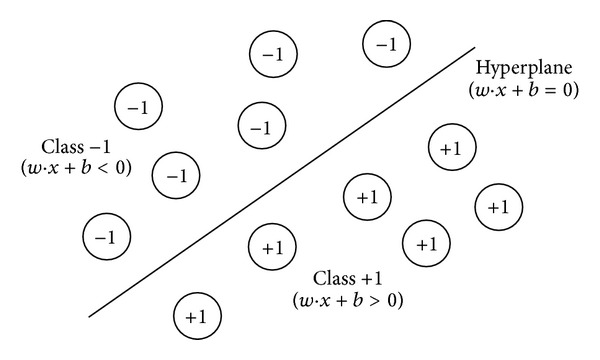
Linear classifier defined by the hyperplane *H* (*w* · *x* + *b* = 0).

A training set of patterns can be separated as linear if there exists at least one linear classifier expressed by the pair (*w*, *b*) which correctly classifies all training patterns as can be seen in Figure 1. This linear classifier is represented by the hyperplane *H* (*w* · *x* + *b* = 0) and defines a region for class +1 patterns (*w* · *x* + *b* > 0) and another region for class −1 patterns (*w* · *x* + *b* < 0).

After the training process, the classifier becomes ready for prediction of the class membership on new patterns, different from training. The class of a pattern *x*_*k*_ is found from the following equation:

(10) $$\begin{array}{c} \text{class}\left(x_{k}\right)=\{\begin{array}{cc} +1 & \text{if}\;w\cdot x_{k}+b>0\\ -1 & \text{if}\;w\cdot x_{k}+b<0. \end{array} \end{array}$$

Thus, the classification of new patterns relies on only the sign of the expression *w* · *x* + *b* [30].

Sequential Minimal optimization is used in the training stage of SVM. SMO algorithm is a popular optimization method used to train the support vector machine (SVM). The dual presentation of an SVM primal optimization problem is indicated in (11):

(11) max⁡αΨ(α)=∑i=1Nαi−12∑i=1N ∑j=1Nyiyjk(xi,xj)αiαjsubject  to∑i=1Nyiαi=0, 0≤αi≤C,  i=1,…,n,

where *x*_*i*_ is a training sample, *y*_*i*_ ∈ {−1, +1} is the corresponding target value, *α*_*i*_ is the Lagrange multiplier, and *C* is a real value cost parameter [31].

### 2.4. Performance Evaluation

Four criteria for performance evaluation of hepatitis, liver disorders and diabetes datasets were used. These criteria are classification accuracy, confusion matrix, analysis of sensitivity and specificity, and *k*-fold cross-validation. 

#### 2.4.1. Classification Accuracy

In this study, the classification accuracies for the datasets are measured with the following the equation:

(12) accuracy(T)=∑i=1Nassess(ti)N, ti∈T,assess(ti)={1,if classify(ti)≡correctclassification,0,otherwise,

where *T* is the classified set of data items (the test set) and *N* is the number of testing samples of the dataset. We will also show the accuracy of our performed *k*-fold cross-validation (CV) experiment.

#### 2.4.2. Confusion Matrix

The confusion matrix includes four classification performance indices: true positive, false positive, false negative, and true negative as given in Table 1. They are also usually used in the two-class classification problem to evaluate the performance.

#### 2.4.3. Analysis of Sensitivity and Specificity

The following expressions were used for calculating sensitivity, specificity, positive predictive value, and negative predictive value; we use [32]:

(13) $$\begin{array}{c} \text{Sensitivity}\;\;\left(\%\right)=\frac{\text{TP}}{\text{TP}+\text{FN}}\times 100,\\ \text{Specificity}\;\left(\%\right)=\frac{\text{TN}}{\text{TN}+\text{FP}}\times 100,\\ \text{Positive predctive value}\;\left(\%\right)=\frac{\text{TP}}{\text{TP}+\text{FP}}\times 100,\\ \text{Negative predicitive value}\;\left(\%\right)=\frac{\text{TN}}{\text{TN}+\text{FN}}\times 100. \end{array}$$

#### 2.4.4. *k*-Fold Cross-Validation

*k*-fold cross-validation is used for the test result to be more valuable [33]. In *k*-fold cross-validation, the original sample is divided into random *k* subsamples, one of which is retained as the validation data for model testing and the remaining *k*-1 sub-samples are used for training The cross-validation process is then repeated *k* times (the folds), with each of the *k* sub-samples used exactly once as the validation data. The process is repeated *k* times (the folds), with each of the *k* sub-samples used only once as the validation data. The average of *k* results from the folds gives the test accuracy of the algorithm [34]. 

## 3. Experimental Work

**Figure 2:**
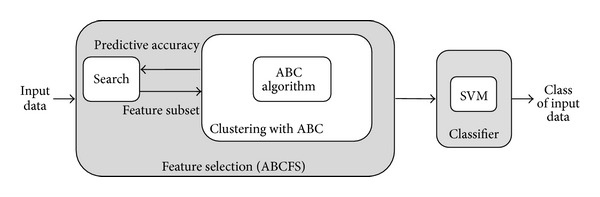
Block diagram of the proposed system.

Less distinctive features of the data set affect the classification negatively. Such data especially decrease the speed and the system performance significantly. With the proposed system, using the feature selection algorithm, the features with less discriminant data were eliminated. The reduced data set increased the testing success of the classifier and the rate of the system. From Figure 2, the proposed system has two phases. At the first phase, as selection criteria, clustering with ABC algorithm was used for feature selection, and, thus, a more effective feature selection method was constituted. Hence, it has been made possible both to select the related features in a shorter period of time and to reduce the dimension of the feature vector. At second stage, the obtained reduced data is supplied to the SVM classifier to determine the accuracy rates. The *k*-fold cross-validation was used for the classifier reliability improvement.

In this study, ABCFS + SVM system is suggested in order to solve the three classification problem named as Hepatitis dataset, Liver Disorders dataset, Diabetes dataset, respectively. 

### 3.1. Datasets

We used the dataset from the UCI machine learning database [35], which is commonly used among researchers for classification, that gives us a chance to compare the performance of our method with others. The datasets of this work can be defined shortly as follows. 

#### 3.1.1. Hepatitis Dataset

This dataset was donated by Jozef Stefan Institute, Yugoslavia. The purpose of the dataset is to predict the presence or absence of hepatitis disease from the different medical tests results of a patient. This database contains 19 attributes. There are 13 binary and 6 discrete values. Hepatitis dataset includes 155 samples from two different classes (32 “die” cases, 123 “live” cases). This dataset contains missing attribute values. We substituted the missing data by frequently encountered values of own class. Attributes of symptoms that are obtained from patient are given in Table 2 [3, 35].

#### 3.1.2. Liver Disorders Dataset

The liver disorders dataset is named as BUPA liver disorders. The liver disorders database includes 6 features, that is, MCV, alkphos, SGPT, SGOT, gammaGT and drinks. There are 345 data in total and each sample is taken from an unmarried man. Two hundred of them are chosen for one class with the remaining 145 are in the other. The first 5 features are all blood tests which are sensitive to liver disorders that arise from excessive alcohol consumption. This dataset is donated by Richard S. Forsyth et al. in 1990. The attributes are given in Table 3 [13].

#### 3.1.3. Diabetes Dataset

This dataset contains 768 samples, where each sample has 8 features which are eight clinical findings. All patients of the dataset are Pima Indian women in which the youngest one is 21 years old and living near Phoenix, Arizona, USA. The binary target variable can take “0” or “1.” If it takes “1,” it means a positive test for Diabetes, or if it takes “0,” it means a negative test. There are 268 different cases in class “1” and 500 different cases in class “0.” The features and parameters are given in Table 4 [16].

### 3.2. Feature Selection with ABC

In the system, a searching process runs to find the best feature subset same like sequential forward selection algorithm. Prediction accuracy for feature selection is found by ABC clustering. Pseudocode of the developed feature selection algorithm based on ABC is given in Pseudocode 3.

**Pseudocode 3:**
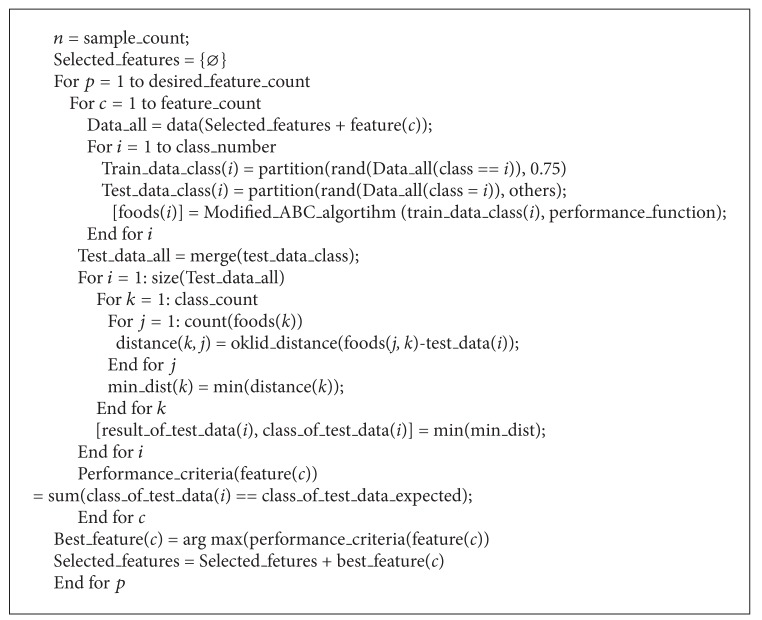
Pseudo-code of developed feature selection algorithm based on ABC.

In Pseudocode 3, *n* is sample count and *p* is desired feature count which is selected as providing the highest performance criteria. While *data* represents the entire dataset, *Data_all* includes the features that are considered chosen. *Train_data_all* is generated by taking 75% of the data found in all classes of *Data_all*. *Test_data_all* is generated by taking 25% of the data that are found in all classes of *Data_all*. Thus, a uniform dispersion was obtained according to the classes. Train data that belongs to each class (*train_data_class*) is trained by the ABC algorithm, which has been modified to cluster. At the end of training, 10 feature vectors named food and representing each class are obtained. Goodness of the chosen feature cluster is described by the food values accuracy representing the test dataset. The error value is found by taking the difference between the test data class and the food value class having a minimum Euclidean distance to the test data class. 

The performance value shows the suitability of the added property. The most appropriate property value does not belong to the chosen properties cluster. This process is repeated by starting from an empty cluster up until the desired feature number. The decline in the value of rising performance trend is for determining the maximum number of features. In summary, in ABCFS, it starts from selecting the feature set as empty, then adds the feature(c) that results in the highest objective function.

We selected colony size 20, maximum cycle/generation number (MCN) 300, and limit value 200. The algorithm was run 100 times. Performance value is found by taking the average of these algorithm results. 

The datasets used for evaluating ABCFS performance and their features are as follows: the number of classes, the number of samples, the number of features and the number of selected features, which are given in Table 5. 

### 3.3. SVM Classification Parameters

The reliability of the classifier was provided by the *k*-fold cross-validation method. While this classifier was used, the training was performed according to the parameters in Table 6. 

## 4. Experimental Results and Discussion

ABCFS + SVM method test results developed for the hepatitis dataset, liver disorders dataset and diabetes datasets are given in Pseudocode 3. These test results contain the classification performance values achieved by the developed methodology by the help of 10-fold cross-validation. The performance values include average classification accuracy, sensitivity, specificity, positive predictive value, and negative predictive value of the proposed system which are given in Table 7. The results of the study show that the average correctness rate of the studies performed so far on all used datasets by employing the method of *k*-fold cross-validation is a very promising result.

For the hepatitis dataset, the comparisons with the other systems are given in Table 8.

For the liver disorders dataset, the comparisons with the other systems are given in Table 9.

For the diabetes dataset, the comparisons with the other systems are given in Table 10.

## 5. Conclusions

This study was designed for use in the diagnosis of liver and diabetes. In these databases that were used, there are some redundant and low-distinctive features. These features are very important factors affecting the success of the classifier and the system processing time. In the system we have developed, the elimination of these redundant features increased the system speed and success. The artificial bee Colony (ABC) algorithm, which is a very popular optimization method, was used for the feature selection process in the study. The ABC-based feature selection algorithm that was developed in this study is the first example of the ABC algorithm used in the field of feature selection. The databases that are subjected to feature selection are classified using SVM. In order to achieve a reliable performance of the classifier, the 10-fold cross-validation method was used. The system results were compared with the literature articles that use the same databases. Classification accuracy of the proposed system reached 94.92%, 74.81%, and 79.29% for hepatitis dataset, liver disorders dataset and diabetes dataset, respectively. Obtained results show that the performance of the proposed method is highly successful compared to other results attained and seems very promising for pattern recognition applications.

## Figures and Tables

**Table 1:** The four classification performance indices included in the confusion matrix.

Actual class	Predicted class	
	Positive	Negative
Positive	True positive (TP)	False negative (FN)
Negative	False positive (FP)	True negative (TN)

**Table 2:** Range values and attribute names for hepatitis dataset [35].

The number of attribute	The name of attribute	Interval of attribute
1	Age	7–78
2	Sex	Male, Female
3	Steroid	No, Yes
4	Antivirals	No, Yes
5	Fatigue	No, Yes
6	Malaise	No, Yes
7	Anorexia	No, Yes
8	Liver big	No, Yes
9	Liver firm	No, Yes
10	Spleen palpable	No, Yes
11	Spiders	No, Yes
12	Ascites	No, Yes
13	Varices	No, Yes
14	Bilirubin	0.3–8
15	Alk phosphate	26–295
16	SGOT	14–648
17	Albumin	2.1–6.4
18	Protime	0–100
19	Histology	No, Yes

**Table 3:** Range values and attribute names for liver disorders dataset [35].

The number of attribute	The name of attribute	Description of the attribute	Interval of attribute
1	MCV	Mean corpuscular volume	65–103
2	Alkphos	Alkaline phosphatase	23–138
3	SGPT	Alamine aminotransferase	4–155
4	SGOT	Aspartate aminotransferase	5–82
5	gammaGT	Gamma-glutamyl transpeptidase	5–297
6	Drinks	Number of half-pint equivalents of alcoholic beverages drunk per day	0–20

**Table 4:** Features and parameters of the diabetes dataset.

Features	Mean	Standard deviation	Min	Max
Number of times pregnant	3.8	3.4	0	17
Plasma glucose concentration, 2 h in an oral glucose tolerance test	120.9	32.0	0	199
Diastolic blood pressure (mm Hg)	69.1	19.4	0	122
Triceps skinfold thickness (mm)	20.5	16.0	0	99
2-hour serum insulin (mu U/mL)	79.8	115.2	0	846
Body mass index (kg/m^2^)	32.0	7.9	0	67.1
Diabetes pedigree function	0.5	0.3	0.078	2.42
Age (years)	33.2	11.8	21	81

**Table 5:** List of datasets.

Databases	Number of classes	Samples	Number of features	Number of selected features	Selected features
Hepatitis	2	155	19	11	12, 14, 13, 15, 18, 1, 17, 5, 16, 2, 4
Liver disorders	2	345	6	5	5, 3, 2, 4, 1
Diabetes	2	768	8	6	2, 8, 6, 7, 4, 5

**Table 6:** List of classification parameters.

Parameters	Value
Method	SVM
Optimization algorithm	SMO
Validation method	*k*-fold cross-validation (10-fold CV)
Kernel_Function	Linear
TolKKT	1.0000*e* − 003
MaxIter	15000
KernelCacheLimit	5000
The initial value	Random

**Table 7:** Performance of classification for the hepatitis, liver disorders, and diabetes datasets.

Performance criteria	Hepatitis dataset	Liver disorders dataset	Diabetes dataset
Classification accuracy (%)	94.92	74.81	79.29
Sensitivity (%)	97.13	88.22	89.84
Specificity (%)	88.33	56.68	59.61
Positive predictive value (%)	96.91	73.99	80.63
Negative predictive value (%)	88.33	78.57	75.65

**Table 8:** Classification accuracies obtained by our method and other classifiers for the hepatitis dataset.

Author (year)	Method	Classification accuracy (%)
Polat and Güneş (2006) [1]	FS-AIRS with fuzzy res. (10-fold CV)	92.59
Polat and Güneş (2007) [2]	FS-Fuzzy-AIRS (10-fold CV)	94.12
Polat and Güneş (2007) [3]	AIRS (10-fold CV)	76.00
	PCA-AIRS (10-fold CV)	94.12
Kahramanli and Allahverdi (2009) [4]	Hybrid system (ANN and AIS) (without *k*-fold CV)	96.8
Dogantekin et al. (2009) [5]	LDA-ANFIS	94.16
Bascil and Temurtas (2011) [6]	MLNN (MLP) + LM (10-fold CV)	91.87
Our study	ABCFS + SVM (10-fold CV)	**94.92**

**Table 9:** Classification accuracies obtained by our method and other classifiers for the liver disorders dataset.

Author (year)	Method	Classification accuracy (%)
Lee and Mangasarian (2001) [7]	SSVM (10-fold CV)	70.33
van Gestel et al. (2002) [8]	SVM with GP (10-fold CV)	69.7
Gonçalves et al. (2006) [9]	HNFB-1 method	73.33
Özşen and Güneş (2008) [10]	AWAIS (10-fold CV)	70.17
	AIS with hybrid similarity measure (10-fold CV)	60.57
	AIS with Manhattan distance (10-fold CV)	60.21
	AIS with Euclidean distance (10-fold CV)	60.00
Li et al. (2011) [11]	A fuzzy-based nonlinear transformation method + SVM	70.85
Chen et al. (2012) [12]	(PSO) + 1-NN method (5-fold CV)	68.99
Chang et al. (2012) [13]	CBR + PSO (train: 75%-test: 25%)	76.81
Our study	ABCFS + SVM (train: 75%-test: 25%)	**82.55**
	ABCFS + SVM (10-fold CV)	**74.81**

**Table 10:** Classification accuracies obtained by our method and other classifiers for diabetes dataset.

Author (year)	Method	Classification accuracy (%)
Şahan et al. (2005) [14]	AWAIS (10-fold CV)	75.87
Polat and Güneş (2007) [15]	Combining PCA and ANFIS	89.47
Polat et al. (2008) [16]	LS-SVM (10-fold CV)	78.21
	GDA-LS-SVM (10-fold CV)	82.05
Kahramanli and Allahverdi (2008) [17]	Hybrid system (ANN and FNN)	84.2
Patil et al. (2010) [18]	Hybrid prediction model (HPM ) with reduced dataset	92.38
Isa and Mamat (2011) [19]	Clustered-HMLP	80.59
Aibinu et al. (2011) [20]	AR1 + NN (3-fold CV)	81.28
Our study	ABCFS + SVM (train: 75%-test: 25%)	**86.97**
	ABCFS + SVM (10-fold CV)	**79.29**
